# The role of indoleamine 2,3-dioxygenase 1 in early-onset post-stroke depression

**DOI:** 10.3389/fimmu.2023.1125634

**Published:** 2023-02-24

**Authors:** Hengshu Chen, Xia Huang, Chang Zeng, Dongren Sun, Fan Liu, Jingyuan Zhang, Qiao Liao, Shihang Luo, Weiye Xu, Yeqing Xiao, Danfeng Zeng, Mingyu Song, Fafa Tian

**Affiliations:** ^1^ Department of Neurology, Xiangya Hospital, Central South University, Changsha, China; ^2^ Department of Critical Care Medicine, The First People’s Hospital of Huaihua, Huaihua, China; ^3^ Health Management Center, Xiangya Hospital, Central South University, Changsha, China; ^4^ Department of Human Anatomy and Neurobiology, School of Basic Medical Sciences, Central South University, Changsha, China; ^5^ Department of Neurology, Hengyang Central Hospital, Hengyang, China; ^6^ Department of Neurology, Xiangtan Central Hospital, Xiangtan, China; ^7^ Department of Clinical Pharmacology, Xiangya Hospital, Central South University, Changsha, China

**Keywords:** indoleamine 2,3-dioxygenase, post-stroke depression, single nucleotide polymorphism, immune-inflammatory response, pro-inflammatory cytokine

## Abstract

**Background:**

The immune-inflammatory response has been widely considered to be involved in the pathogenesis of post-stroke depression (PSD), but there is ambiguity about the mechanism underlying such association.

**Methods:**

According to Diagnostic and Statistical Manual of Mental Disorders (5th edition), depressive symptoms were assessed at 2 weeks after stroke onset. 15 single nucleotide polymorphisms (SNPs) in genes of indoleamine 2,3-dioxygenase (IDO, including IDO1 and IDO2) and its inducers (including pro-inflammatory cytokines interferon [IFN]-γ, tumor necrosis factor [TNF]-α, interleukin [IL]-1β, IL-2 and IL-6) were genotyped using SNPscan™ technology, and serum IDO1 levels were detected by double-antibody sandwich enzyme-linked immune-sorbent assay.

**Results:**

Fifty-nine patients (31.72%) were diagnosed with depression at 2 weeks after stroke onset (early-onset PSD). The IDO1 rs9657182 T/T genotype was independently associated with early-onset PSD (adjusted odds ratio [OR] = 3.008, 95% confidence interval [CI] 1.157-7.822, *p* = 0.024) and the frequency of rs9657182 T allele was significantly higher in patients with PSD than that in patients with non-PSD (χ2 = 4.355, *p* = 0.037), but these results did not reach the Bonferroni significance threshold (*p* > 0.003). Serum IDO1 levels were also independently linked to early-onset PSD (adjusted OR = 1.071, 95% CI 1.002-1.145, *p* = 0.044) and patients with PSD had higher serum IDO1 levels than patients with non-PSD in the presence of the rs9657182 T allele but not homozygous C allele (t = -2.046, *p* = 0.043). Stroke patients with the TNF-α rs361525 G/G genotype had higher serum IDO1 levels compared to those with the G/A genotype (Z = -2.451, *p* = 0.014).

**Conclusions:**

Our findings provided evidence that IDO1 gene polymorphisms and protein levels were involved in the development of early-onset PSD and TNF-α polymorphism was associated with IDO1 levels, supporting that IDO1 which underlie strongly regulation by cytokines may be a specific pathway for the involvement of immune-inflammatory mechanism in the pathophysiology of PSD.

## Introduction

There is increasingly robust evidence that the activation of immune-inflammatory pathways plays an etiological role in the development and progression of post-stroke depression (PSD) (1), a complex and common post-stroke complication associated with increased morbidity and mortality (2), although its exact pathogenesis remains undetermined. The involvement of inflammation in the pathophysiology of PSD was initially based on an inflammatory hypothesis (3, 4) in which acute stroke induces a wide spectrum of central and peripheral immune-inflammatory responses, accompanied by upregulation of various pro-inflammatory cytokines (interleukin [IL]-1β, tumor necrosis factor [TNF]-α, interferon [IFN]-γ and IL-6, for instance) (5), subsequently resulting in increased expression of the gene encoding enzyme indoleamine 2,3-dioxygenase (IDO) 1 that triggers the depletion of serotonin, a unanimously identified feature of depression (6). Increasing data have since proven that pro-inflammatory cytokines can act as biomarkers of PSD. Su et al. found that TNF-α, IFN-γ and IL-6 levels were elevated in patients suffering from PSD within 1 year after stroke (7). In addition, Kim et al. observed that high serum levels of TNF-α and IL-1β were associated with an increased risk of PSD, especially in the acute stage of stroke and in patients carrying susceptible genes (8). Additionally, results from Kang et al. demonstrated that higher serum IL-18 levels were independently related to PSD in the early and chronic phase after stroke (9), in line with the investigation by Yang et al. showing the predictive role of IL-18 levels in the risk of PSD (10). Despite the robust association of pro-inflammatory cytokines with the occurrence of PSD, it is unclear whether the effect of increased immune activation resulting from stroke on the risk of PSD is associated with IDO1 expression.

IDO1 is an enzyme strictly regulated by cytokines and can be expressed in a variety of cells throughout the body in response to immunological signals, including IFN-γ, TNF-α, IL-1β, IL-2, and IL-6 stimulation (6, 11). It has been well-established that IDO1 plays an important role in the etiology of depression through two mechanisms (6, 12), one is that the overactivated IDO1, an initial and key rate-limiting enzyme of the tryptophan catabolite pathway, tends to direct tryptophan down the kynurenine pathway that releases quinolinic acid, a powerful N-methyl-D-aspartate (NMDA) receptor agonist with definite neurotoxic effects which is involved in the onset of depression (13, 14), and the other is that IDO1 shunts tryptophan from the serotonin synthesis route, thereby favoring depression (15). These observations indicate that IDO1 activation is relatively unique to inflammation-induced depression (16). The available results, furthermore, reveal that upregulation of IDO1 activation is a characteristic of the post-stroke inflammatory response (17, 18). Based on these findings, IDO1 may be a pivotal mediator of the contribution of stroke-associated inflammatory processes to the development of PSD. As such, the present study was designed to investigate the association between gene polymorphisms of IDO (IDO1 and IDO2, a recently recognized enzyme structurally and functionally similar to IDO1 (19)) and its inducers (including IFN-γ, TNF-α, IL-1β, IL-2 and IL-6) and PSD at 2 weeks after stroke (early-onset PSD), taking into account the role of serum IDO1 levels in the pathophysiology of early-onset PSD. In parallel, we analyzed the association between cytokine SNPs and serum IDO1 levels.

## Materials and methods

### Study population and clinical assessment

186 acute stroke patients hospitalized at the Department of Neurology, Xiangya Hospital of Central South University were recruited from July 2019 to February 2021. Inclusion criteria were: (1) age from 18-75 years, (2) diagnosed with acute stroke by brain magnetic resonance imaging or computerized tomography imaging within 2 weeks since onset, (3) ability to complete all necessary evaluations. The exclusion criteria were performed as described previously (20). Written informed consents were signed from all patients, as approved by Medical Ethics Committee of the Xiangya Hospital of Central South University. We collected the information on demographic data (age, gender and years of education), vascular risk factors (hypertension, diabetes, heart disease, hyperlipidemia, current smoking and drinking), history of stroke, transient ischemic attack (TIA), intravenous thrombolysis and/or endovascular treatment, type of stroke (ischemic, hemorrhagic or subtypes according to the Trial of Org 10,172 in Acute Stroke Treatment [TOAST] classification) (21), stroke hemisphere (left, right or bilateral) and location (anterior, posterior or both), National Institute of Health Stroke Scale (NIHSS) score and Mini-Mental State Examination (MMSE) score, time from stroke onset to the blood sample collection, the complete blood counts (leukocyte, neutrophil, monocyte, lymphocyte and platelet counts) from the first blood routine results, pulmonary and/or urinary tract infection, and antibiotic. And the assessment of depressive symptoms and grouping of patients have been described in our previous investigation (20).

### Gene polymorphism selection and genotyping

We identified 15 SNPs in genes of IDO (IDO1 and IDO2) and its inducers (including IFN-γ, TNF-α, IL-1β, IL-2 and IL-6) selected from previous literature associated with PSD or depression or stroke, with a minor allele frequency > 0.05 indexed in the Chinese Han population dataset of a genetic database (http://www.ensembl.org). Each SNP was genotyped using SNPscan™ multiple SNP genotyping technology (22) unaware of the sample status.

### Measurement of serum IDO1 levels

Fasting venous blood samples were obtained within 2 weeks after stroke onset. Quantitative IDO1 assay was performed for the determination of IDO1 concentrations in serum using double-antibody sandwich enzyme-linked immune-sorbent assay kit provided by Shanghai Tianhao Biotechnology Co., Ltd., China. The minimum detectable dose was less than 0.1 ng/mL. The inter- and intra-assay coefficients of variation were less than 10% and 15%, respectively.

### Statistical analysis

The data was analyzed using the IBM SPSS Statistics for Windows, version 26 (IBM Corp, Armonk, NY, USA). Shapiro-Wilk test was used to determine the normality of continuous variables. Continuous variables with normal distribution were summarized as means ± standard deviations assessed by Student’s t-test, and continuous variables with non-normal distribution were presented as median (interquartile range [IQR]) analyzed by Mann-Whitney U test. Categorical variables were reported as absolute number (percentage value) compared by Chi-squared test or Fisher’s exact test. Hardy-Weinberg equilibrium (HWE) was examined using the Chi-squared test based on the genotype distribution in non-PSD group. The binary logistic regression model allowing adjustment for statistically significant confounding factors, in addition, was performed to identify independent risk factors for PSD. A two-side *p* value of less than 0.05 was considered statistically significant and the multiple comparisons were adjusted by the Bonferroni correction with a corrected *p*-value threshold (*p* = 0.05/15 = 0.003), given that fifteen tests were performed for the association of each SNP with PSD. Furthermore, depending on data distribution, the correlation between cytokine SNPs and serum IDO1 levels was evaluated using one-way analysis of variance with Bonferroni *post-hoc* test or non-parametric test (Kruskal-Wallis or Mann-Whitney U test) (p < 0.05).

## Results

### Demographic and clinical characteristics

General demographic and clinical characteristics of the cohort were shown in **Table 1**. In the participants as a whole, a median (IQR) age of them was 57 (51–65) years and 57 (30.65%) were female, 15 (8.06%) of whom were diagnosed with hemorrhagic stroke. PSD was found in 59 (31.72%) patients who experienced an assessment of depressive symptoms along with non-PSD patients on Day 14 after stroke onset. Compared to the non-PSD group, the PSD group exhibited a higher distribution of female (40.68% versus 25.98%, *p* = 0.043), lower median (IQR) of years of education (9 [6-12] versus 12 [9-12], *p* = 0.018), and lower MMSE score reflecting cognitive function (24 [21-28] versus 26 [23-29], *p* = 0.033), but none of them were independently associated with PSD status (**Table 2**). No statistically significant differences, however, were observed between the PSD and non-PSD groups with respect to age, vascular risk factors, history of stroke, TIA, intravenous thrombolysis and/or endovascular treatment, type of stroke, stroke hemisphere and location, NIHSS score, time from stroke onset to the blood sample collection, the complete blood counts from the first blood routine results, pulmonary and/or urinary tract infection, and antibiotic.

**Table 1:** General demographic and clinical profiles between PSD and non-PSD groups.

Variables	Non-PSD (n=118)	PSD (n=52)	*P* value
Age (years)	57 (51–65)	58 (52-65)	0.506
Female	33 (25.98)	24 (40.68)	0.043
Years of education	12 (9-12)	9 (6-12)	0.018
Vascular risk factors			
Hypertension	88 (69.29)	42 (71.19)	0.793
Diabetes	32 (25.20)	20 (33.90)	0.218
Heart disease	10 (7.87)	4 (6.78)	1.000
Hyperlipidemia	47 (37.01)	22 (37.29)	0.971
Current smoking	61 (48.03)	27 (45.76)	0.773
Current drinking	70 (55.12)	27 (45.76)	0.235
History of stroke	10 (7.87)	4 (6.78)	1.000
TIA	12 (9.45)	5 (8.48)	0.830
IVT and/or EVT	17 (13.39)	6 (10.17)	0.535
Stroke type: ischemic stroke	118 (92.91)	53 (89.83)	0.564
TOAST classification			0.768
Large-artery atherosclerosis	82 (69.49)	42 (79.25)	
Small-vessel occlusion	15 (12.71)	5 (9.43)	
Cardioembolism	7 (5.93)	3 (5.66)	
Other determined etiology	4 (3.39)	1 (1.89)	
Undetermined etiology	10 (8.48)	2 (3.77)	
AIS hemisphere			0.105
Left	57 (44.88)	20 (33.90)	
Right	57 (44.88)	36 (61.01)	
Bilateral	13 (10.24)	3 (5.09)	
AIS location			0.855
Anterior	70 (55.12)	35 (59.32)	
Posterior	46 (36.22)	19 (32.20)	
Both	11 (8.66)	5 (8.48)	
NIHSS score	2 (1-5)	3 (1-8)	0.062
MMSE score	26 (23-29)	24 (21-28)	0.033
Onset to blood sample collection (days)	3 (1-5)	3 (1-7)	0.427
Leukocyte count (10^9^/L)	7.0 (5.9-8.1)	7.0 (5.6-8.3)	0.771
Neutrophil count (10^9^/L)	4.5 (3.7-5.8)	4.6 (3.6-5.8)	0.847
Monocyte count (10^9^/L)	0.5 (0.4-0.6)	0.5 (0.4-0.6)	0.366
Lymphocyte count (10^9^/L)	1.5 (1.2-2.1)	1.5 (1.2-1.7)	0.958
Platelet count (10^9^/L)	201 (172-242)	198 (172-228)	0.546
PI and/or UTI	25 (19.69)	11 (18.64)	0.867
Antibiotic	16 (12.60)	6 (10.17)	0.633
IDO1 (ng/mL)	25.90 ± 4.91	27.51 ± 5.23	0.043

Values are absolute number (percentage value), median (interquartile range) or means ± standard deviations. PSD, post-stroke depression; TIA, transient ischemic attack; IVT, intravenous thrombolysis; EVT, endovascular treatment; TOAST, Trial of Org 10,172 in Acute Stroke Treatment; AIS, acute ischemic stroke; NIHSS, National Institutes of Health and Stroke Scale; MMSE, Mini-Mental State Examination; IDO1, indoleamine 2,3-dioxygenase 1; PI, pulmonary infection; UTI, urinary tract infection.

**Table 2:** Independent risk factors for early-onset PSD.

Variables	Unadjusted		Adjusted	
	OR (95%CI)	*P* value	OR (95%CI)	*P* value
IDO1 rs9657182				
C/C	Ref	–	Ref	–
C/T	1.472 (0.642-3.371)	0.361	1.412 (0.598-3.334)	0.431
T/T	2.615 (1.048-6.529)	0.039	3.008 (1.157-7.822)	0.024
IDO1 (ng/mL)	1.067 (1.002-1.137)	0.045	1.071 (1.002-1.145)	0.044
Gender	1.953 (1.016-3.755)	0.045	1.442 (0.658-3.160)	0.361
Years of education	0.901 (0.823-0.987)	0.025	0.945 (0.847-1.054)	0.311
MMSE score	0.940 (0.880-1.004)	0.066	0.962 (0.896-1.033)	0.290

PSD, post-stroke depression; OR, odds ratio; CI, confidence interval; IDO1, indoleamine 2,3-dioxygenase 1; MMSE, Mini-Mental State Examination.

### Genotype and allelic frequencies by PSD status

The genotype and allele frequencies of IDO (IDO1 and IDO2) and its inducers (including IFN-γ, TNF-α, IL-1β, IL-2 and IL-6) SNPs in PSD and non-PSD patients were summarized in **Table I in the Supplementary Material**. There was no deviation from HWE for any genotype in non-PSD group (all *p* > 0.05). As shown in **Table I**, the genotype and allele distributions of IDO1 polymorphisms in PSD group were distinct from those in patients with non-PSD, showing that the frequency of the IDO1 rs9657182 T/T genotype was significantly higher in PSD patients than non-PSD patients before (odds ratio [OR] = 2.615, 95% confidence interval [CI] 1.048-6.529, *p* = 0.039) and after adjusting potential confounders including gender, years of education, MMSE score and serum IDO1 levels (adjusted OR = 3.008, 95% CI 1.157-7.822, *p* = 0.024) (**Table 2**), and that the frequency of the rs9657182 T allele was also significantly higher in patients with PSD compared to those with non-PSD (χ2 = 4.355, *p* = 0.037), but these differences disappeared after Bonferroni correction (*p* > 0.003). We did not observe any significant differences in either the genotype or allelic frequencies of IDO1 rs7820268 and IDO2 rs2929115 between PSD and non-PSD groups, as did gene polymorphisms of IDO inflammatory stimuli (including IFN-γ, TNF-α, IL-1β, IL-2 and IL-6). According to the foregoing, the IDO1 rs9657182 T/T genotype was independent risk factor of early-onset PSD and the rs9657182 T allele conferred an elevated risk for the development of early-onset PSD, providing suggestive association of the IDO1 rs9657182 polymorphism with the risk of early-onset PSD (0.003 < P < 0.05).

### Association of serum IDO1 levels with PSD status

**Figure 1:**
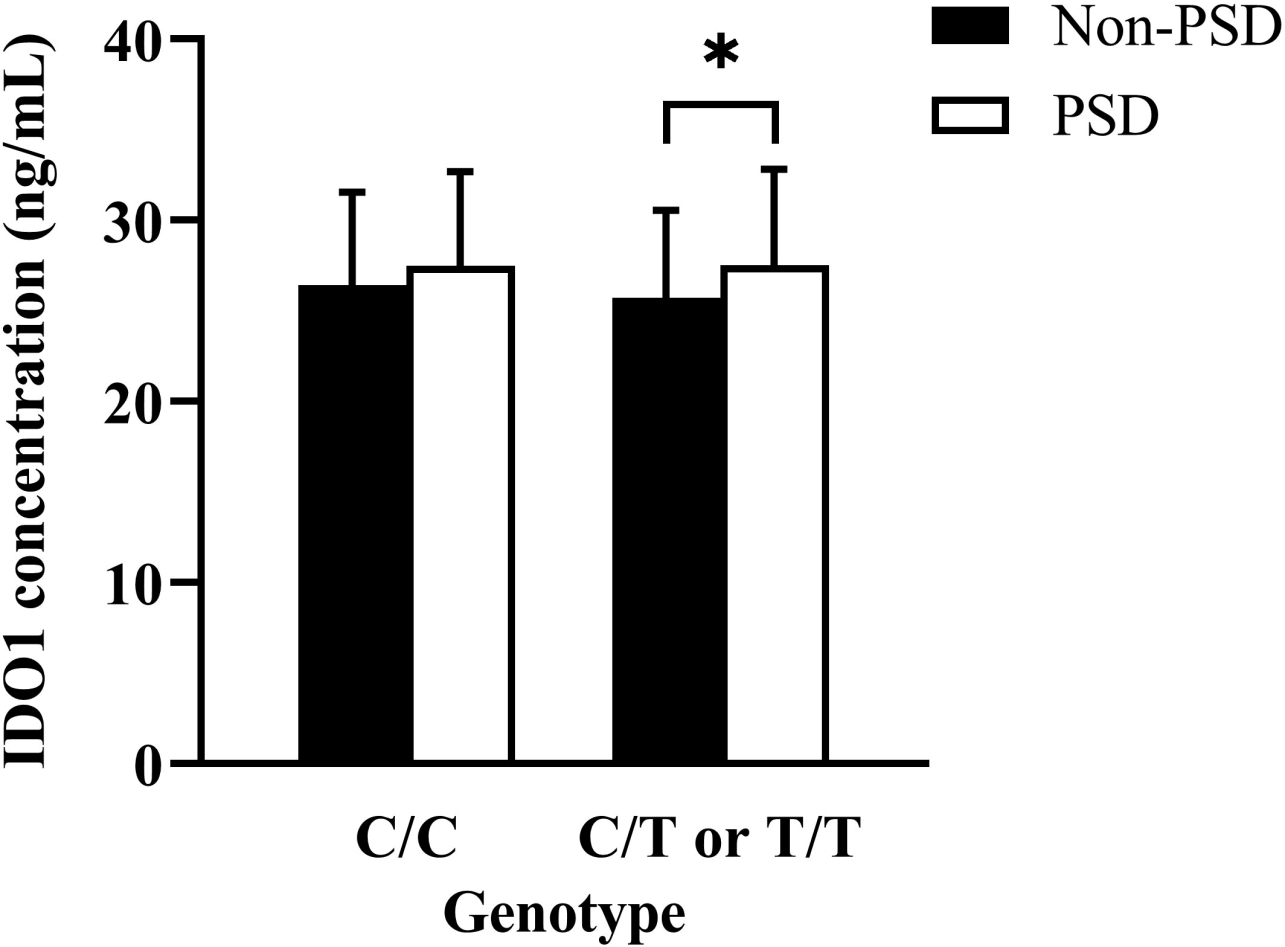
Comparison of serum indoleamine 2,3-dioxygenase (IDO) 1 concentrations according to the presence or absence of the IDO1 rs9657182 T allele between PSD and non-PSD patients. The result was presented as median (interquartile range) on the left side of **Figure 1** compared by the Mann-Whitney U-test, and the data was shown as means ± standard deviations on the right side of **Figure 1** compared by the Student’s t-test. *p < 0.05.

In the study population, in comparison to patients with non-PSD, serum IDO1 levels were higher in patients with PSD (t = -2.040, *p* = 0.043) (**Table 1**), suggesting that the increased serum IDO1 levels were related to an elevated risk of early-onset PSD. Moreover, the association with PSD status remained stable even after controlling for possible covariates shown in **Table 2** (adjusted OR = 1.071, 95% CI 1.002-1.145, *p* = 0.044). Additionally, serum IDO1 levels were further analyzed depending on the IDO1 rs9657182 allele distribution and PSD status, and we found that patients with PSD showed greater serum IDO1 levels than patients with non-PSD in the presence of the rs9657182 T allele but not homozygous C allele (t = -2.046, *p* = 0.043) (**Figure 1**). These results indicated that there was an independent association between serum IDO1 levels and early-onset PSD, and that the IDO1 rs9657182 T allele increased the risk of early-onset PSD by enhancing serum IDO1 levels.

### Association of serum IDO1 levels with cytokine SNPs

Serum IDO1 levels of the study population by gene polymorphisms of pro-inflammatory cytokines including IFN-γ, TNF-α, IL-1β, IL-2 and IL-6 were listed in **Table II in the Supplementary Material**. Serum IDO1 levels were significantly higher in stroke patients carrying the TNF-α rs361525 G/G genotype than in those carrying the G/A genotype (Z = -2.451, *p* = 0.014), indicating that TNF-α rs361525 polymorphism was associated with serum IDO1 levels. Besides, there was a correlation between TNF-α rs1799964 polymorphism and serum IDO1 levels (F = 3.564, *p* = 0.030) but in adjusted Bonferroni *post-hoc* comparisons this finding did not reach the threshold of statistical significance (T/T versus T/C genotype, *p* = 0.076; T/T versus C/C genotype, *p* = 0.297; T/C versus C/C genotype, *p* = 1.000). No significant relationships of serum IDO1 levels with SNPs of IFN-γ, IL-1β, IL-2 and IL-6 were found (*p* > 0.05).

## Discussion

The present study examined for the first time the role of gene polymorphisms of IDO (IDO1 and IDO2) and its inducer and serum IDO1 levels in the risk of PSD at 2 weeks after stroke onset and also discussed the relationship between cytokine SNPs and serum IDO1 levels. The results preliminarily indicated here that IDO1 rs9657182 T allele was a suggestive predisposing factor for early-onset PSD, which was associated with increased serum IDO1 levels possibly due to the T allele affecting the transcriptional activity of the promoter region of the IDO1 gene, and that patients with acute stroke who carry the rs9657182 T/T genotype had an increased susceptibility to early-onset PSD, and that serum IDO1 levels were an independent risk factor for early-onset PSD, and that serum IDO1 levels in stroke settings were relevant to the TNF-α rs361525 polymorphism. These findings provided further support for the cytokine hypothesis of PSD during the acute phase of stroke and also suggested that the rs9657182 polymorphism and serum IDO1 levels might be novel diagnostic biomarkers and/or intervention targets for early-onset PSD.

Considering the overwhelming evidence on a responsible role of pro-inflammatory cytokines in the etiology of PSD (1) and cytokine-inducible IDO1 as a key factor in inflammation-induced depression (23), gene polymorphisms involved in determining the functional activity of IDO1 and its inducer cytokines are promising candidate contributors to PSD. In this regarding, gene polymorphisms of pro-inflammatory cytokines that stimulate IDO1 expression, including IFN-γ, TNF-α, IL-1β, IL-2 and IL-6, were analyzed between PSD and non-PSD patients. The result here that SNPs in genes encoding the above IDO-associated inflammatory stimulants were not correlated with early-onset PSD was in accordance with our previous study showing that there was no link between gene polymorphisms of pro-inflammatory cytokines (IFN-γ, TNF-α, IL-1β and IL-6) and early-onset PSD (20). Furthermore, Kim et al. also found that pro-inflammatory cytokines, such as TNF-α, IL-1β and IL-6, were independent of depression during the acute phase of stroke (24). A previous investigation, however, highlighted the potential synergistically effects of TNF-α and IL-1β, considering their corresponding alleles together, on the risk of PSD at 2 weeks post-stroke (8). These inconsistent findings may be related to the following events: the observability of functional polymorphisms of pro-inflammatory cytokines is influenced by population distribution (24) and there is heterogeneity in respect to diagnostic criteria of depression, sample size and subject selection.

In parallel, the association of the rs9657182 polymorphism, localized in the promoter region of the IDO1 gene (25), with PSD status was also explored in present study. Although polymorphisms in these pro-inflammatory cytokines were not risk factors for early-onset PSD status, we observed that the IDO1 rs9657182 polymorphism was correlated with early-onset PSD, providing suggestive evidence for a potential causal link between the IDO1 polymorphism and the risk of early-onset PSD. Specifically, the T/T genotype of IDO1 rs9657182 investigated in this study was an independent precipitating factor for early-onset PSD and the rs9657182 T allele was also a risk factor for it, similar to the finding of Smith et al. suggesting that the rs9657182 polymorphism was a predictor of the development of cytokine-induced depressive symptoms during treatment with IFN-α in Caucasian patients with chronic hepatitis C (25). The data derived from animal models, besides, displayed that inflammatory stimuli did not induce depression-like behavior when IDO1 gene was genetically deficient (26). These investigations highlighted the causative role of IDO1 polymorphism in the pathophysiology of depression mediated by immune stimulation. However, there was no association between early-onset PSD and the rs2929115 polymorphism of IDO2, adjacent to IDO1 gene and with a similarity of IDO2 to IDO1 in protein structure (27), indicating either that IDO2 has limited correlation with early-onset PSD or that the primary acts of IDO2 lie outside of its enzymatic function (28). And we further assessed the relevance of serum IDO1 levels to early-onset PSD status. Our study demonstrated that the correlation of serum IDO1 levels with early-onset PSD varied by the rs9657182 allele distribution. Increased serum IDO1 levels in stroke patients carrying the T allele but not homozygous C allele of the rs9657182 conveyed a liability to early-onset PSD, and in combination with the evidence on the contribution of the T allele to early-onset PSD formation, it could be assumed that the polymorphism in the IDO1 gene promoter region at position rs9657182 affected the expression levels of IDO1 in response to cytokines stimulation following stroke, giving rise to the development of early-onset PSD. It was interesting to note that serum IDO1 levels were associated with an increased risk of early-onset PSD, independent of the presence of the IDO1 rs9657182 polymorphism, possibly because IDO1 production was subject to the complex interplay and/or synergy of numerous parameters in immune-inflammatory settings, such as other IDO1 gene polymorphisms and multiple pro-inflammatory cytokine concentrations. Our finding was further supported by a preclinical study that denoted increased IDO expression in the nucleus accumbens, hippocampus, and hypothalamus of PSD-like phenotype mice (29). There are several underlying mechanisms that may account for the association of IDO1 with PSD: the elevated levels of pro-inflammatory cytokines after stroke upregulate IDO1 expression that causes the depletion of serotonin precursor tryptophan and increased neurotoxic kynurenine metabolite (quinolinic acid), an agonist of NMDA receptors, leading to the occurrence of depression (14, 30–32). IDO1 has been seen as a central hub linking immune-inflammatory processes to the monoaminergic (33) and glutamatergic systems implicated in depression (14). Our results, taken together, supported that the interactions of cytokine- serotonin and -glutamate *via* IDO1 could play a key role in PSD (3, 4).

In the state of stroke-induced immune activation, upon analysis on the relationship between individual genetic variation in cytokines and circulating IDO1 levels, we observed that stroke patients harboring the G/G genotype at the rs361525 locus of the TNF-α gene had higher serum IDO1 levels compared to the G/A genotype. We hypothesized that the association of serum IDO1 levels with the rs361525 polymorphism was related to the expression of TNF-α. The possible explanation is that the SNP rs361525 in the promoter region of TNF-α gene enhance the production of TNF-α (34, 35), which synergistically promotes IDO1 upregulation with other cytokines (36, 37). It should be noted that in our cases there was no mutant homozygous A allele of the rs361525. More credible large-scale studies are warranted to clarify the role of the TNF-α levels in combination with its gene polymorphism in IDO1 expression, especially in the context of immune activation. Interestingly for our purposes, the TNF-α rs361525 polymorphism may be indirectly involved in the development of early-onset PSD, given the findings in the present study that the rs361525 polymorphism was associated with serum IDO1 levels, which were an independent risk factor for early-onset PSD. Our data, collectively, provided reasonable grounds to assume that IDO1 may be a crucial mediator linking inflammation and early-onset PSD.

The limitations of the current study need to be considered. Firstly, this cross-sectional study contributed to establishing a preliminary association between IDO1 and early-onset PSD, but it would be informative to design longitudinal studies to assess the value of IDO1 in late-onset PSD. Secondly, although there is a degree of overlap between brain and peripheral IDO1 activity (23), results based on serum IDO1 levels could not be readily applied to the central nervous system and their relationship needs to be specifically investigated. Thirdly, due to the limitation of sample size, we did not further evaluate the severity of PSD and it could be interesting to understand if there is an association between gene polymorphisms, serum IDO1 levels and severity of depression in a larger study population. Fourthly, serum levels of IDO inflammatory stimulants, including IFN-γ, TNF-α, IL-1β, IL-2 and IL-6, were not measured and the combination of cytokine levels with their corresponding gene polymorphisms helps to identify the optimal risk factors for PSD.

In conclusion, our study had shed light on that gene polymorphisms as suggestive risk predictors and expression levels of IDO1 were involved in depression occurring during the acute stage of stroke and the TNF-α rs361525 polymorphism was associated with serum IDO1 levels. These findings may be a meaningful addition to the neuroimmune pathways in the pathophysiology of early-onset PSD and serum IDO1 levels, alone or in combination with its corresponding polymorphism, may allow for a different intervention focusing on more specific etiologically-based management for early-onset PSD. Future adequately powerful trials are necessary to elucidate the role of IDO1 associated with the post-stroke immune-inflammatory responses in the pathogenesis of PSD, especially depression during the acute phase of stroke, from different perspectives.

## Data availability statement

The original contributions presented in the study are included in the article/**Supplementary Materials**. Further inquiries can be directed to the corresponding authors.

## Ethics statement

The studies involving human participants were reviewed and approved by Medical Ethics Committee of the Xiangya Hospital of Central Department of Clinical Pharmacology, Xiangya Hospital, Central South University, Changsha, China South University.

## Author contributions

HC, XH, DS, FL, MS and FT contributed to conception and design of the study. HC, XH, JZ, QL and MS organized the database. HC wrote the first draft of the manuscript. HC, CZ, SL, WX, YX, DZ wrote sections of the manuscript. All authors contributed to the article and approved the submitted version.
