# Supplementary material for: The role of indoleamine 2,3-dioxygenase 1 in early-onset post-stroke depression
Source: Front Immunol. 2023 Feb 24;14:1125634. doi: 10.3389/fimmu.2023.1125634 (PMC9998486; doi:10.3389/fimmu.2023.1125634)
Supplement: Supplementary file 1 [file DataSheet_1.pdf]

Supplementary Material

Supplementary Tables

Table I Genotype and allele distributions in PSD and non-PSD patients.

Gene	SNP	Genotype or allele	Non-PSD, n (%)	PSD, n (%)	OR (95%CI) or χ^2	*P* value
IDO1	rs7820268	C/C	77 (60.63)	34 (57.63)	Ref	-
		C/T	44 (34.65)	18 (30.51)	0.926 (0.469-1.830)	0.826
		T/T	6 (4.72)	7 (11.86)	2.642 (0.826-8.451)	0.101
		C	198 (77.95)	86 (72.88)	1.147	0.284
		T	56 (22.05)	32 (27.12)		
IDO1	rs9657182	C/C	34 (26.77)	10 (16.95)	Ref	-
		C/T	67 (52.76)	29 (49.15)	1.472 (0.642-3.371)	0.361
		T/T	26 (20.47)	20 (33.90)	2.615 (1.048-6.529)	0.039
		C	135 (53.15)	49 (41.52)	4.355	0.037
		T	119 (46.85)	69 (58.48)		
IDO2	rs2929115	A/G	1 (0.79)	1 (1.70)	Ref	-
		G/G	126 (99.21)	58 (98.30)	0.460 (0.028-7.488)	0.586
		A	1 (0.39)	1 (0.85)	-	0.534
		G	253 (99.61)	117 (99.15)		
IFN-γ	rs1861494	C/C	13 (10.24)	8 (13.56)	Ref	-
		C/T	53 (41.73)	26 (44.07)	0.797 (0.294-2.162)	0.656
		T/T	61 (48.03)	25 (42.37)	0.666 (0.246-1.803)	0.424

Gene	SNP	Genotype or allele	Non-PSD, n (%)	PSD, n (%)	OR (95%CI) or χ^2	*P* value
		C	79 (31.10)	42 (35.59)	0.740	0.390
		T	175 (68.90)	76 (64.41)		
TNF-α	rs1799724	C/C	99 (77.95)	45 (76.27)	Ref	-
		C/T	26 (20.47)	11 (18.64)	0.931 (0.423-2.047)	0.858
		T/T	2 (1.58)	3 (5.09)	3.300 (0.533-20.440)	0.199
		C	224 (88.19)	101 (85.59)	0.492	0.483
		T	30 (11.81)	17 (14.41)		
TNF-α	rs1799964	T/T	96 (75.59)	42 (71.19)	Ref	-
		T/C	27 (21.26)	14 (23.73)	1.185 (0.565-2.485)	0.653
		C/C	4 (3.15)	3 (5.08)	1.714 (0.367-7.998)	0.493
		T	219 (86.22)	98 (83.05)	0.642	0.423
		C	35 (13.78)	20 (16.95)		
TNF-α	rs1800629	G/G	111 (87.40)	55 (93.22)	Ref	-
		G/A	15 (11.81)	4 (6.78)	0.538 (0.171-1.699)	0.291
		A/A	1 (0.79)	0 (0.00)	-	-
		G	237 (93.31)	114 (96.61)	1.650	0.199
		A	17 (6.69)	4 (3.39)		
TNF-α	rs361525	G/G	123 (96.85)	56 (94.92)	Ref	
		G/A	4 (3.15)	3 (5.08)	1.647 (0.357-7.607)	0.523
		G	250 (98.43)	115 (97.46)	-	0.684
		A	4 (1.57)	3 (2.54)		

Gene	SNP	Genotype or allele	Non-PSD, n (%)	PSD, n (%)	OR (95%CI) or χ^2	*P* value
IL-1β	rs1143623	C/C	43 (33.86)	16 (27.12)	Ref	-
		C/G	58 (45.67)	27 (45.76)	1.251 (0.601-2.605)	0.549
		G/G	26 (20.47)	16 (27.12)	1.654 (0.709-3.858)	0.244
		C	144 (56.69)	59 (50.00)	1.456	0.228
		G	110 (43.31)	59 (50.00)		
IL-1β	rs1143627	G/G	33 (25.98)	20 (33.90)	Ref	-
		G/A	59 (46.46)	26 (44.07)	0.727 (0.353-1.497)	0.387
		A/A	35 (27.56)	13 (22.03)	0.613 (0.263-1.426)	0.256
		G	125 (49.21)	66 (55.93)	1.456	0.228
		A	129 (50.79)	52 (44.07)		
IL-1β	rs1143634	G/G	122 (96.06)	59 (100.00)	Ref	-
		G/A	5 (3.94)	0 (0.00)	-	-
		G	249 (98.03)	118 (100.00)	-	0.183
		A	5 (1.97)	0 (0.00)		
IL-1β	rs1143643	C/C	30 (23.62)	21 (35.59)	Ref	-
		C/T	63 (49.61)	24 (40.68)	0.544 (0.262-1.129)	0.102
		T/T	34 (26.77)	14 (23.73)	0.588 (0.255-1.357)	0.213
		C	123 (48.42)	66 (55.93)	1.817	0.178
		T	131 (51.58)	52 (44.07)		
IL-2	rs2069762	A/A	53 (41.73)	28 (47.46)	Ref	-
		A/C	59 (46.46)	23 (38.98)	0.738 (0.380-1.434)	0.370
		C/C	15 (11.81)	8 (13.56)	1.010 (0.382-2.670)	0.985

Gene	SNP	Genotype or allele	Non-PSD, n (%)	PSD, n (%)	OR (95%CI) or χ^2	*P* value
		A	165 (64.96)	79 (66.95)	0.141	0.707
		C	89 (35.04)	39 (33.05)		
IL-6	rs1800795	C/G	0 (0.00)	1 (1.70)	Ref	-
		G/G	127 (100.00)	58 (98.30)	-	-
		C	0 (0.00)	1 (0.85)	-	0.317
		G	254 (100.00)	117 (99.15)		
IL-6	rs2069824	T/T	127 (100.00)	59 (100.00)	-	-

Values are absolute number (percentage value) or median (interquartile range). PSD, post-stroke depression; SNP, single nucleotide polymorphism; OR, odds ratio; CI, confidence interval; IDO1, indoleamine 2,3-dioxygenase 1; IFN-γ, interferon-γ; TNF-α, tumor necrosis factor-α; IL-1β, interleukin-1β.

Table II Serum IDO1 levels by cytokine polymorphisms.

Gene	SNP	Genotype	N	Serum IDO1 levels (ng/mL)	F or H or Z	*P* value
IFN-γ	rs1861494	C/C	21	25.18±4.29	0.841	0.433
		C/T	79	26.36±5.13		
		T/T	86	26.77±5.16		
TNF-α	rs1799724	C/C	144	26.58 (22.12-29.65)	1.561	0.458
		C/T	37	26.66 (22.56-31.74)		
		T/T	5	28.73 (23.62-33.35)		
TNF-α	rs1799964	T/T	138	26.97±4.94	3.564	0.030
		T/C	41	24.97±5.28		
		C/C	7	23.77±3.82		

Gene	SNP	Genotype	N	Serum IDO1 levels (ng/mL)	F or H or Z	*P* value
TNF-α	rs1800629	G/G	166	27.00 (22.49-30.06)	2.493	0.287
		G/A	19	24.99 (21.47-28.77)		
		A/A	1	25.59		
TNF-α	rs361525	G/G	179	26.72 (22.63-30.02)	-2.451	0.014
		G/A	7	20.75 (17.98-26.70)		
IL-1β	rs1143623	C/C	59	26.46±5.22	0.658	0.519
		C/G	85	26.75±4.99		
		G/G	42	25.66±4.98		
IL-1β	rs1143627	G/G	53	25.69±5.27	0.761	0.469
		G/A	85	26.74±4.79		
		A/A	48	26.62±5.29		
IL-1β	rs1143634	G/G	181	26.66 (22.31-29.97)	-0.114	0.909
		G/A	5	25.59 (23.36-29.51)		
IL-1β	rs1143643	C/C	51	25.83±5.15	0.521	0.595
		C/T	87	26.53±4.97		
		T/T	48	26.82±5.16		
IL-2	rs2069762	A/A	81	26.57±4.86	0.753	0.473
		A/C	82	25.99±5.20		
		C/C	23	27.38±5.27		
IL-6	rs1800795	C/G	1	32.35	-1.425	0.154
		G/G	185	26.65 (22.34-29.84)		
IL-6	rs2069824	T/T	186	26.66 (22.35-29.95)	-	-

Values are absolute number, median (interquartile range) or means ± standard deviations. SNP, single nucleotide polymorphism; IDO1, indoleamine 2,3-dioxygenase 1.
